# Functional recovery is considered the most important target: a survey of dedicated professionals

**DOI:** 10.1186/2047-0525-3-5

**Published:** 2014-07-30

**Authors:** Eirik K Aahlin, Maarten von Meyenfeldt, Cornelius HC Dejong, Olle Ljungqvist, Kenneth C Fearon, Dileep N Lobo, Nicolas Demartines, Arthur Revhaug, Stephen J Wigmore, Kristoffer Lassen

**Affiliations:** 1 Department of GI and HPB Surgery, University Hospital Northern Norway, Breivika, Tromsø, Norway; 2 Department of Surgery, University Hospital Maastricht and NUTRIM School for Nutrition, Toxicology and Metabolism,, Maastricht, The Netherlands; 3 Department of Surgery, Örebro University Hospital, Örebro, Sweden; 4 Department of Molecular Medicine and Surgery, Karolinska Institutet, Stockholm, Sweden; 5 Clinical Surgery, University of Edinburgh, Royal Infirmary of Edinburgh, Edinburgh, UK; 6 Division of Gastrointestinal Surgery, Nottingham Digestive Diseases Centre National Institute for Health Research, Biomedical Research Unit, Nottingham University Hospitals, Queen's Medical Centre, Nottingham, UK; 7 Department of Visceral Surgery, University Hospital of Lausanne (CHUV), Lausanne, Switzerland; 8 Institute of Clinical Medicine, University of Tromsø, Tromsø, Norway

**Keywords:** Recovery, Perioperative care, ERAS, Fast track

## Abstract

**Background:**

The aim of this study was to survey the relative importance of postoperative recovery targets and perioperative care items, as perceived by a large group of international dedicated professionals.

**Methods:**

A questionnaire with eight postoperative recovery targets and 13 perioperative care items was mailed to participants of the first international Enhanced Recovery After Surgery (ERAS) congress and to authors of papers with a clear relevance to ERAS in abdominal surgery. The responders were divided into categories according to profession and region.

**Results:**

The recovery targets ‘To be completely free of nausea’, ‘To be independently mobile’ and ‘To be able to eat and drink as soon as possible’ received the highest score irrespective of the responder's profession or region of origin. Equally, the care items ‘Optimizing fluid balance’, ‘Preoperative counselling’ and ‘Promoting early and scheduled mobilisation’ received the highest score across all groups.

**Conclusions:**

Functional recovery, as in tolerance of food without nausea and regained mobility, was considered the most important target of recovery. There was a consistent uniformity in the way international dedicated professionals scored the relative importance of recovery targets and care items. The relative rating of the perioperative care items was not dependent on the strength of evidence supporting the items.

## Background

The Enhanced Recovery After Surgery (ERAS) Critical Pathway concept is based upon a protocol of care items applied perioperatively to achieve optimal stress reduction following surgery with subsequent reductions in overall morbidity and accelerated recovery [1]. The traditional concept dictates that all protocol items are of importance and synergistic effects on recovery are optimal when all items are implemented [1-3]. Recovery, however, is a compound term demanding the fulfilment of several indicators of well-being and is not clearly defined. At the same time, implementing complex interventions like unabridged ERAS protocols is challenging and dependent on smooth cooperation of all groups of health professionals involved in patient care. If health workers from different regions and professions express significantly different views on what recovery implies and on the relative importance of the various ERAS protocol items in achieving this recovery, this may severely undermine successful implementation at ward and institution levels.

Francis D Moore defined surgical convalescence in 1958 in the following way: ‘Convalescence includes all the interlocking physical, chemical, metabolic, and psychological factors commencing with the injury, or even slightly before the injury, and terminating only when the individual has returned to normal physical well-being, social and economic usefulness, and psychological habitus.’ [4]. Whilst FD Moore focused on the importance of functional recovery, length of hospital stay (LOS) has been the dominant outcome in convalescence research in later years [5].

We wanted to investigate how international experts and dedicated professionals view surgical convalescence today. We aimed to investigate differences and similarities on the views on central recovery targets and care items amongst the different professions and backgrounds.

## Methods

### Questionnaire

A questionnaire was developed by the authors specifically for this survey and consisted of two sets of questions:

• Set 1: The responders were asked to score eight different recovery targets on a scale from 1 to 11, where 1 was *not important* and 11 was *very important*.

• Set 2: The responders were also asked to score 13 care items on a similar scale according to the items' perceived importance in achieving recovery.

The targets and items are presented in Tables 1 and 2. The questionnaire was prepared for digital, web-based distribution by email (QuestBack®) and mailed to recipients between October and December 2012.

**Table 1:** Target for recovery

**Target for recovery**	**Mean (SD)**
To be completely free of nausea (not feeling or being sick)	9.88 (1.42)
To be independently mobile in hospital as soon as possible	9.72 (1.53)
To be able to eat and drink as soon as possible	9.59 (1.75)
To be able to return to all daily activities as soon as possible	9.28 (2.10)
To be completely free of pain at rest	9.27 (2.02)
To be completely free of pain upon movement	8.56 (2.8)
To be discharged from hospital as soon as possible	8.30 (2.28)
To be able to move the bowels as soon as possible	8.28 (2.61)

Mean rating of the recovery targets amongst all responders, listed in descending order of importance (scale 1 = not important, 11 = most important).

**Table 2:** Perioperative care item

**Care item**	**Mean (SD)**
Optimizing fluid balance	10.28 (1.22)
Preoperative counselling by nurse, anaesthetist and surgeon	10.26 (1.10)
Promoting early and scheduled mobilisation	10.24 (1.19)
Avoiding nasogastric tube after the operation	9.71 (1.86)
Allowing normal diet at will after the operation	9.70 (1.49)
Antimicrobial prophylaxis and skin preparation	9.62 (1.97)
Preoperative fasting kept at absolute minimum	9.41 (2.08)
Oral carbohydrate loading preoperatively	8.75 (2.40)
Stimulation of gut mobility	8.65 (2.35)
Avoiding a wound drain	8.56 (2.49)
Avoiding oral bowel preparation preoperatively	8.53 (2.45)
Using epidural analgesia for approximately 48 h postop	8.44 (2.78)
Avoiding preanaesthetic sedative medication	8.14 (2.57)

Mean rating of the care items amongst all responders, listed in descending order of importance (scale 1 = not important, 11 = most important).

### Participants

*To reach potential opinion leaders in the respective surgical communities*, we targeted two select groups of professionals:

•Group 1: Delegates to the first international ERAS congress in Cannes, France, in October 2012 were identified from the delegate list and subsequently contacted by email with the attached web-based questionnaire.

• Group 2: A PubMed search was conducted on 13 October 2012 with the following search terms: (‘enhanced recovery’ OR ‘critical pathway’ OR ‘fast track’) AND (‘surgery’ OR ‘operation’ OR ‘resection’ OR ‘*ectomy’), for the latest 5 years in English. The resulting list of papers was hand searched to yield only papers with a clear relevance to ERAS in abdominal surgery. The first and last authors of the papers identified received a mail with the questionnaire, provided they were not already included in group 1.

### Selection of recovery targets and care items

The eight recovery targets and 13 care items have been central in Enhanced Recovery guidelines [6-8] and consensus documents [9] and were chosen after consensus amongst the two senior authors.

Statistical analysis was performed with the SPSS (version 20) statistical package using purely descriptive statistics with calculation of mean score and standard deviation. Non-responders received two reminders per email. The recipients were asked their nationality and divided into regions according to UN definitions ( http://unstats.un.org/unsd/methods/m49/m49regin.htm) and profession (physicians and nurses).

### Ethical approval

This survey of attitudes of medical professionals did not affect patients or other individuals under treatment. The Regional Committee for Medical and Health Research Ethics did not consider formal ethical approval to be required.

## Results

Emails with questionnaires were sent to a total of 311 individuals. In total, we received 165 responses, 121 congress participants and 44 authors. The response rate was 50% from both congress participants and authors. The responders were 103 men and 62 women. There were 87 surgeons, 30 anaesthetists, 28 nurses and 20 respondents with various backgrounds (administrators, dietitians, etc.). The responders' nationalities are presented in Table 3. Of the responders, 68% work primarily with colorectal surgery. The mean score of the recovery targets are presented in Table 1 and the mean score of the care items in Table 2. In both tables, the alternatives are presented in descending order of importance as scored by the responders.

**Table 3:** Nationality

**Region**	**Responders, *n *** **(%)**
Northern Europe	91 (55.2)
Western Europe	22 (13.3)
Eastern and Southern Europe	18 (10.9)
America	19 (11.5)
Asia, Africa and Oceania	15 (9.1)
Total	165

The responders' nationality, divided into regions according to UN definitions.

### Targets for recovery

Two targets had the highest mean score in all responder categories. These were ‘To be completely free of nausea’ and ‘To be independently mobile’. Together with a third target, ‘To be able to eat and drink as soon as possible’, these three targets for recovery were consistently amongst the four targets with the highest mean score irrespective of professional or geographical responder categories. ‘To be able to return to all daily activities as soon as possible’ received the third highest score amongst the nurses (Table 4).

**Table 4:** The highest ranked recovery targets

**Physicians (*n*** **= 117)**	**Nurses**	**Europe**	**The world outside Europe**
	**(*n*** **= 28)**	**(*n*** **= 131)**	**(*n*** **= 34)**
To be completely free of nausea (not feeling or being sick)	To be completely free of nausea (not feeling or being sick)	To be completely free of nausea (not feeling or being sick)	To be independently mobile in hospital as soon as possible
To be independently mobile in hospital as soon as possible	To be independently mobile in hospital as soon as possible	To be independently mobile in hospital as soon as possible	To be completely free of nausea (not feeling or being sick)
To be able to eat and drink as soon as possible	To be able to return to all daily activities as soon as possible	To be able to eat and drink as soon as possible	To be able to eat and drink as soon as possible

The three highest ranked recovery targets, according to profession and geographical region.

### Relative importance of care item to achieve recovery

The following three care items were amongst the four care items with the highest mean score in all responder categories: ‘Optimizing fluid balance’, ‘Preoperative counselling’ and ‘Promoting early and scheduled mobilisation’. The first two care items had the highest mean score in all categories. ‘Avoiding nasogastric tube after the operation’ received the third highest score amongst the responders from the world outside Europe (Table 5).

**Table 5:** The highest ranked perioperative care items

**Physicians**	**Nurses**	**Europe**	**The world outside Europe**
**(*n*** **= 117)**	**(*n*** **= 28)**	**(*n*** **= 131)**	**(*n*** **= 34)**
Optimizing fluid balance	Preoperative counselling by nurse, anaesthetist and surgeon	Optimizing fluid balance	Preoperative counselling by nurse, anaesthetist and surgeon
Preoperative counselling by nurse, anaesthetist and surgeon	Promoting early and scheduled mobilisation	Promoting early and scheduled mobilisation	Optimizing fluid balance
Promoting early and scheduled mobilisation	Optimizing fluid balance	Preoperative counselling by nurse, anaesthetist and surgeon	Avoiding nasogastric tube after the operation

The three highest ranked perioperative care items, according to profession and geographical region.

## Discussion

The recovery targets which received the highest score according to importance in all groups of responders in our survey were ‘To be completely free of nausea’, ‘To be independently mobile’ and ‘To be able to eat and drink as soon as possible’. Amongst the 13 protocol care items listed, ‘Optimizing fluid balance’, ‘Preoperative counselling’ and ‘Promoting early and scheduled mobilisation’ were rated as those most important to achieve this recovery.

The present survey shows how targets for recovery and the relative importance of protocol items are perceived by a large international group of enhanced recovery protocol experts and dedicated professionals. Recruited from delegates to the first international ERAS conference and from principal authors of ERAS-related research literature, many of the responders were likely to be opinion leaders in their local and national perioperative pathway environment. As such, they constitute a body of experience and knowledge that probably reflects the current views held by health workers in a wider context. This is especially interesting when dealing with items where robust evidence is wanting. Our response rate is barely within acceptable limits in surveys of this kind [10], although a higher response rate would have been preferable.

The central finding is the similarities between different professions and regions in terms of scoring the most important recovery targets and care items. This could result from a *de facto* agreement or from bias in a sample of respondents drawn from similar backgrounds. This similarity is not statistically tested. Nevertheless, as stated above, this still indicates uniformity in how the relative importance of recovery targets and protocol care items are perceived and hence the attention this will receive in everyday practice across nations. Several international studies have documented incomplete implementation of various evidence-based Enhanced Recovery protocol items, such as avoidance of oral bowel preparation and nasogastric tube [11-13]. Whilst being long-standing core elements of Enhanced Recovery protocols, they are not consistently top rated in our survey.

‘To be completely free of nausea’, ‘To be independently mobile’ and ‘To be able to eat and drink as soon as possible’ are the recovery targets considered most important by the responders. Another survey amongst international experts concluded that a patient is ready for discharge when ‘there is tolerance of oral intake, recovery of lower gastrointestinal function, adequate pain control with oral analgesia, ability to mobilize and self-care, and no evidence of complications or untreated medical problems’ [14]. This is also consistent with the definition of a recovered patient, ready for discharge, used in both the earliest Enhanced Recovery studies and more recent ones [15,16]. Functional recovery, as in tolerance of food without nausea and regained mobility, is consistently considered the most important target for recovery and might be used when defining the recovered patient in future research and audits.

In a recent publication, recovery was divided into three distinct phases: the early (from the postoperative care unit to the ward), intermediate (from the ward to discharge) and late (from discharge to return to normal function) phases [17]. To be free of nausea, independently mobile and being able to eat and drink might serve as a common target for recovery in the intermediate phase. This is also consistent with the thinking of one of the pioneers in surgical recovery research, FD Moore, who divided recovery into four phases: the injury, the turning point, spontaneous nitrogen anabolism and fat redeposition [4]. ‘The turning point’ corresponds to the intermediate phase of surgical recovery, and the way FD Moore describes this phase has striking similarities to the most important targets for recovery in our survey: ‘…There is an increase in gastrointestinal function, with a return of peristalsis, the passage of flatus by the rectum, a desire for food….’ [4]. The late phase of recovery, when the patient is discharged and struggle to regain normal function has received little attention in research and publications [5]. This phase might be especially important to patients and society, and it deserves to be investigated in studies with adequate follow-up length.

Interestingly, and somewhat surprising, was the finding that three of the targets and items which consistently received the highest score: ‘Prevention of nausea’, ‘Preoperative counselling’ and ‘Early mobilisation’, were the only items (together with *audit*) that were not supported by a Grade A recommendation in the 2009 ERAS consensus guidelines for colorectal surgery [9]. The level of evidence supporting these three targets and items was also considered low or very low in the latest guidelines for perioperative care in elective pancreaticoduodenectomy, rectal/pelvic surgery and colonic surgery [6-8]. They were, however, strongly recommended [6-8].

This indicates that experts and dedicated professionals' views on target and/or care item importance are not necessarily linked to the level of evidence supporting it, as was also repeatedly the case in the recent guidelines [6-8]. Other surveys have shown that surgeons tend to have higher confidence in their own judgement than all other resources [18,19]. Surveys have also shown that there are frequent misconceptions about central terms in evidence-based medicine within the surgical community, like misconceptions concerning important aspects of evidence hierarchy and common terminology in study design [20]. In the era of evidence-based medicine, this serves as a reminder of the fact that there is no absolute relationship between perceived importance and established evidence.

Most targets and items were rated as important, indicating that our questionnaire lacks discriminatory power. However, any responder would be likely to think that one cannot have too much of a good thing and hence be unlikely to rate any target or item as ‘not important’. Our data should be complemented with patients' ratings on the same targets and items. This would add to our understanding of the causes that impede successful implementation of protocols.

Our survey was intended to be a short mapping of the relative importance of central recovery targets and care items today as perceived by the international expertise. This could serve as a basis for more formal research aimed at creating common definitions of the different phases of recovery. Such research might include the Delphi methodology, both in the selection of targets and items and in the further research process [21].

## Conclusions

There was a striking uniformity in the way international expertise scored the relative importance of recovery targets and protocol items, and this rating was not dependent on the strength of supporting evidence. Functional recovery, as in tolerance of food without nausea and regained mobility, was considered the most important target for recovery and might be used as a definition of intermediate recovery in future research and audits. One definition of recovery that covers all phases and aspects might not be possible or even desirable.

## Competing interests

All authors are members of the ERAS society. The authors declare that they have no other competing interests. No grant support was received.

## Authors' contributions

EKA contributed to the study concepts, study design, data acquisition, data analysis and interpretation, quality control of data and algorithms, manuscript preparation, manuscript editing and manuscript review. MvM, CHCD, OL, KCF, DNL, ND and SJW contributed to the study concepts, study design, quality control of data and algorithms, manuscript editing and manuscript review. AR contributed to the study concepts, study design, quality control of data and algorithms, manuscript editing, manuscript preparation and manuscript review. KL contributed to the study concepts, study design, data acquisition, quality control of data and algorithms, manuscript preparation, manuscript editing and manuscript review. All authors read and approved the final manuscript.
